# When and why do people act on flawed science? Effects of anecdotes and prior beliefs on evidence-based decision-making

**DOI:** 10.1186/s41235-021-00293-2

**Published:** 2021-04-06

**Authors:** Audrey L. Michal, Yiwen Zhong, Priti Shah

**Affiliations:** Department of Psychology, University of Michigan, 530 Church St., Ann Arbor, MI 48109 USA

**Keywords:** Anecdotes, Evidence evaluation, Scientific reasoning, Education, Prior beliefs

## Abstract

Today’s citizens are expected to use evidence, frequently presented in the media, to inform decisions about health, behavior, and public policy. However, science misinformation is ubiquitous in the media, making it difficult to apply research appropriately. Across two experiments, we addressed how anecdotes and prior beliefs impact readers’ ability to both identify flawed science and make appropriate decisions based on flawed science in media articles. Each article described the results of flawed research on one of four educational interventions to improve learning (Experiment 1 included articles about having a tidy classroom and exercising while learning; Experiment 2 included articles about using virtual/augmented reality and napping at school). Experiment 1 tested the impact of a single anecdote and found no significant effect on either participants’ evidence evaluations or decisions to implement the learning interventions. However, participants were more likely to adopt the more plausible intervention (tidy classroom) despite identifying that it was unsupported by the evidence, suggesting effects of prior beliefs. In Experiment 2, we tested whether this intervention effect was driven by differences in beliefs about intervention plausibility and included two additional interventions (virtual reality = *high plausible*, napping = *low plausible*). We again found that participants were more likely to implement *high plausible* than *low plausible* interventions, and that evidence quality was underweighed as a factor in these decisions. Together, these studies suggest that evidence-based decisions are more strongly determined by prior beliefs than beliefs about the quality of evidence itself.

## Introduction

Suppose you came across an article on a popular website titled “*Knowing Your Learning Style Can Help You Succeed in School and Beyond*.” Would you change the way you learn information based on that article? Would you send that article to your family and friends? In fact, according to a recent survey of nearly 3000 people, 90% of participants reported believing that people learn better when information is presented in their preferred learning modality (i.e., visual, auditory, read/written, or kinesthetically; Boser, 2017). However, despite the pervasive belief in its effectiveness, the learning styles theory is considered a ‘neuromyth’ that has virtually no evidence in support of it (e.g., Kirschner, & van Merriënboer, 2013; Nancekivell et al., 2020; Pashler et al., 2008).

While the example above is specific to education, people are increasingly expected to apply scientific findings to real-world problems. Some of these decisions are personal, such as deciding whether to consume genetically modified foods or vaccinate your children. Decisions can also take place in a work context; teachers or administrators might decide which curricula to adopt, physicians decide which medications to prescribe, and so forth. The prevalence of making decisions based on scientific claims, or evidence-based decision-making, is thus growing across several fields, from medicine, public health, and education to everyday life.

Yet some popular press articles are touting fake science news, which may include pseudoscientific claims (e.g., ‘effectiveness’ of homeopathic remedies), exaggerated headlines (e.g., “*Young people are growing horns from cellphone use: study”)*, and endorsements of bad science, such as the retracted 1998 *Lancet* article showing a supposed link between MMR vaccines and autism (Eggertson, 2010). Additionally, science news articles may oversell scientific claims (e.g., Bromme & Goldman, 2014), for instance by not including hedging language or citing a single study as conclusive rather than putting it in the context of a body of literature. In part because of the speed and accessibility of social media, the spread of health and science misinformation is ubiquitous (e.g., Kouzy et al., 2020; Merchant & Asch, 2018; Sharma et al., 2017). Critically, the spread of science misinformation can lead to dangerous health consequences, from people misusing hydroxychloroquine, bleach and other disinfecting products to treat COVID-19 symptoms (Gharpure et al., 2020; American Association of Poison Control Centers, 2020) to parents refusing to vaccinate their children (McCauley et al., 2012).

In a perfect world, people would critically and objectively evaluate the existing evidence for a given recommendation, then either adopt recommendations that have solid empirical support or reject recommendations that are not supported by the existing evidence. Furthermore, there are at least two circumstances under which people should reject a given recommendation based on scientific claims: when the evidence in *support* of a claim is of *low quality* (i.e., it is based on fake science claims, including pseudoscience, bad science, oversold claims, etc.), or when the evidence *refuting* a claim is of *high quality* (e.g., meta-analyses showing a small effect size, multiple failures to replicate, etc.). Here we focus on the first scenario: how well can people evaluate fake science news, and to what extent does poor-quality evidence inform decisions in the presence of other factors, such as endorsements from others and prior beliefs?

In practice, people struggle to distinguish between low- and high-quality scientific evidence, particularly in popular press contexts. Pseudoscientific claims may be especially compelling because of ‘illusions of causality,’ in which people tend to infer causal relationships when none exists because of a general causality bias (Matute et al., 2011). In a similar vein, people often accept correlational data as evidence of causality in science media reports (e.g., Burrage, 2008; Robinson & Levin, 2019; Rodriguez, Ng, et al., 2016). Flaws in experimental design such as low sample size or invalid measurement are rarely noticed spontaneously (Burrage, 2008; Rodriguez, Ng, et al., 2016). More generally, people may not always be able to detect oversold scientific claims, whether those claims come from the researchers themselves or the reporting journalist (e.g., Bromme & Goldman, 2014). Furthermore, superficial factors impact evidence evaluation, often causing low-quality evidence to appear more compelling. For example, Weisberg et al. (2008) found that people were more likely to accept bad psychological explanations when irrelevant neuroscience was included (Weisberg et al., 2008; see also Beck (2010); Fernandez-Duque et al., 2015; Rhodes et al., 2014; Weisberg et al., 2015; Hopkins et al., 2016; Im et al., 2017). People are also more persuaded by low-quality scientific claims that are accompanied by anecdotes (Rodriguez, Rhodes, et al., 2016) and endorsement cues, such as a greater number of Facebook ‘likes’ (Luo et al., 2020), as well as prior exposure to misinformation (Pennycook et al., 2018).

In particular, the presence of anecdotal evidence can serve as a powerful barrier for scientific reasoning and evidence-based decision-making. Anecdotal evidence generally conveys narrative information, including personal stories and testimonies (Kazoleas, 1993). A substantial body of work has shown that people are more persuaded by anecdotal than statistical evidence (e.g., Tversky and Kahneman, 1974; Borgida & Nisbett, 1977; although see Hornikx (2005) for an alternative perspective). For instance, Borgida and Nisbett (1977) found that decisions among undergraduates about which future courses to take were influenced by anecdotal recommendations from a handful of other students, but not by more informative statistical evidence (i.e., mean course evaluations from hundreds of peers). The influence of anecdotal evidence in decision-making has even been observed among practitioners in evidence-based fields such as health care (e.g., Fagerlin et al., 2005; Lomas et al., 1991) and education (Blackman et al., 2018; Koballa, 1986). Several mechanisms have been proposed to explain the persuasive power of anecdotes, such as their increased vividness (e.g., Herr et al., 1991) and emotional appeal (e.g., Small et al., 2007), as well as people’s belief in the ‘law of small numbers’ (Tversky & Kahneman, 1971), which could account for peoples’ tendency to generalize from the experiences of a small sample (Borgida & Nisbett, 1977). In other words, people tend to overestimate the representativeness of just a few anecdotal examples and underweigh more reliable consensus information provided by a much larger group. Thus, even when the data in support of a claim are reliable, people’s decisions may be more influenced by anecdotes both because anecdotes are overvalued and because statistical information is undervalued.

Knowing how anecdotal evidence factors into people’s decisions is particularly important in the context of evaluating science news, because people typically must consider both anecdotal and statistical evidence simultaneously (e.g., Hornikx, 2018; Jaramillo et al., 2019). How do people weigh both anecdotal and scientific evidence when judging claims and making decisions? For instance, patients may struggle to decide whether to take medical advice from close relatives and friends or follow evidence-based recommendations from their physician (e.g., Enkin & Jadad, 1998; Fagerlin et al., 2005; Kosko, 2006). Consistent with previous work, anecdotal evidence appears to dominate reasoning and decision-making in these scenarios, even when people are given the opportunity to consider (and comprehend) scientific evidence and base-rate information (e.g., Hornikx, 2018; Jaramillo et al., 2019). People are also less likely to attend to scientific and statistical evidence in the presence of anecdotes (e.g., Fagerlin et al., 2005; Rodriguez, Rhodes, et al., 2016); for example, when reading about fictitious scientific findings, the presence of anecdotes decreased the likelihood that people detected methodological errors and increased the persuasiveness of the flawed studies (Rodriguez, Rhodes, et al., 2016).

While the presence of extraneous factors such as anecdotes may have an influence on reasoning about evidence, it is important to note that prior beliefs have an even more robust effect. Much prior research has established that people are more critical of belief-inconsistent evidence compared to belief-consistent evidence (Lord et al., 1979; Koehler, 1993; for a relatively recent review, see Shah et al., 2017). A dual-process model explanation for this phenomenon is that people process belief-consistent information in a more heuristic manner, but take a more analytic approach to evaluating belief-inconsistent information (Klaczynski, 2000; Kunda, 1990; Stanovich & West, 2000). In particular, Evans and colleagues propose that when people encounter evidence that they agree with, they activate the default, heuristic mode of thinking. Encountering evidence inconsistent with their beliefs triggers the activation of the analytic system (e.g., Thompson et al., 2012). In some cases, this motivated critique can lead to appropriate rejection of bad science. At the same time, motivated reasoning might actually promote rejection of scientific evidence that is widely accepted by experts, as in the context of climate change (Lewandowsky & Oberauer, 2016).

In either case, however, it is unclear to what extent evidence-based decisions are based on critical evaluations of evidence in contexts that are strongly belief-consistent or belief-inconsistent. In particular, people tend to have strong prior beliefs about what works well in education, even if those beliefs are largely incorrect, as in the case of the learning styles theory (e.g., Boser, 2017). To what extent can flawed evidence influence peoples’ decisions to reject an educational intervention that they already believe to be effective? In the studies presented here, we attempted to control for prior beliefs by providing participants with the identical science studies about educational interventions (either with or without an anecdote). However, there may still be an effect of prior beliefs about different educational interventions, and it is also possible that anecdotal evidence could interact with prior beliefs (e.g., anecdotes might be more influential when people don’t already hold strong prior beliefs about a topic).

In Experiment 1, similar to Rodriguez, Rhodes, et al., 2016, we were interested in whether the presence of anecdotes would affect how people evaluate fake science news about two potential educational interventions; thus, we predicted that the presence of anecdotes would decrease readers’ attention to the quality of evidence with clear flaws and lead to inflated ratings of evidence strength. Based on prior work showing that educators are more likely to base teaching decisions on anecdotes from peers and colleagues than scientific evidence (e.g., Blackman et al., 2018), a new question we wanted to address was whether anecdotes would affect evidence-based decisions when the evidence was of low quality; specifically, we predicted that the presence of anecdotes would increase the likelihood that people would adopt a recommendation based on bad science. To foreshadow, the anecdotes did not have a significant effect on participants’ evidence quality ratings or likelihood of adopting an intervention. However, we found an inconsistency such that participants preferred to implement the intervention that was supported by a study rated as more flawed compared to the intervention that was supported by a less flawed study. Follow-up analyses revealed that the two interventions differed substantially in their plausibility, and that participants were more likely to implement the more plausible intervention despite recognizing that the supporting evidence for it was weak. Experiment 2 tested whether this unexpected finding from Experiment 1 was replicable, whether it extended to a broader set of examples, and its underlying mechanism.

## Experiment 1

### Method

In the first experiment, we sought to replicate the findings of Rodriguez, Rhodes, et al. (2016) in the context of fake science news about two potential educational interventions. Specifically, we examined whether including personal anecdotes would decrease participants’ ability to evaluate low-quality evidence presented in a popular press context. Additionally, we tested whether the presence of personal anecdotes would influence participants’ decisions about whether to implement an educational intervention in a hypothetical classroom.

#### Participants

87 undergraduate students (44 females, 32 males, 11 not recorded) were recruited from the University of Michigan Introductory Psychology Subject Pool. This number of participants was chosen to be consistent with a similar study (Rodriguez, Rhodes, et al., 2016), whose effects we were trying to replicate. The average age of the students was 18 years, ranging from 17 to 21. Student participants were granted half an hour of credit for participating. All participants consented to participate in the study, which was approved by the University of Michigan Institutional Review Board.

#### Procedure

Participants completed the study either online or in-person, and all materials were presented on a computer using Qualtrics software (Qualtrics, Provo, UT). Participants were instructed to read two fictitious media articles (A and B), each describing a research study about a potential educational intervention. There were two versions of each article: one with an anecdote (e.g., article A +) and one without an anecdote (e.g., e.g., article A). The presentation order and the article version were randomized, such that each participant read one article with an anecdote and the other article without an anecdote. Participants were thus randomly assigned to four possible conditions: 1) article A + , article B; 2) article A, article B + ; 3) article B + , article A; 4) article B, article A + . After reading each article, participants completed a comprehension check by responding to one multiple choice question about the article. We next measured participants’ evaluation of each article (using measures based on Macpherson & Stanovich, 2007) using a Likert scale (1 to 5). Participants were asked to rate the strength of the evidence for the researcher’s claim (e.g., “How would you rate the strength of evidence for the researcher's claim that exercise aids language learning?; 1 = ‘very weak’, 5 = ‘very strong’), the persuasiveness of the study (1 = ‘very unpersuasive’, 5 = ‘very persuasive’) and the likelihood that participants would implement the technique discussed in the article in a hypothetical class setting (e.g., ‘Imagine that you are a middle school teacher teaching a second language. Based on this study, how likely is it that you would incorporate physical exercise into your lessons?’; 1 = ‘very unlikely’, 5 = ‘very likely’). We also asked participants to explain their reasoning using an open-ended response for the evidence rating (e.g., ‘Why did you rate the evidence for the researcher's claim as weak/strong?’) and likelihood of implementing rating (e.g., ‘Why would you choose/not choose to incorporate physical exercise into your lessons?’). At the end of the survey, participants provided basic background information, including gender, age and the highest level of statistics class taken.

#### Materials

The media articles used in this study were fictional and described research studies on the effectiveness of two educational interventions—learning while exercising and learning in a tidy classroom (see "Appendix 1" for example articles). The exercise intervention article was adopted from a previous study showing that exercising while studying improves second language learning compared to not exercising (Liu et al., 2017), while the tidy classroom intervention was fictional and showed that taking a test in a tidy room boosted academic performance compared to an untidy classroom. The articles were designed to resemble an online popular press article, including a headline, the author’s name, date, a generic picture related to the intervention, and a one-page long article. The articles started with a brief introduction or an anecdotal story related to the study, followed by a brief description of the research study. All versions of the articles (with/without anecdote, exercise/tidy classroom study) were made roughly the same length to rule out the possibility that longer articles would increase persuasiveness. We deliberately planted experimental flaws in the scientific methods (i.e., procedures, type of control group, validity of measures) and errors in interpretation of results in all articles. There were three main categories of design flaws, including non-random assignment/sampling bias, other types of confounds and invalid measures. Specifically, in the description of the exercise study, participants were assigned to exercise or control groups according to their preferences (non-random assignment) and performance was measured via self-report instead of quantitatively (invalid measure). In the description of the tidy classroom study, the participant groups were unequal, such that half of the participants came from math class and half came from English class before taking a math exam (participant confound), and participants were primed to believe that being in a messy room might hurt their performance before taking the exam (priming confound).

In the anecdote versions of the articles, the anecdote consisted of a single story that favored the new teaching intervention. For the exercise intervention article, the story featured two Chinese boys learning English as a second language, with one boy who exercised while studying outperforming the other boy, who did not exercise while studying, on an English vocabulary test. For the tidy classroom intervention article, the story was about a boy whose messy desk negatively impacted his mood and interfered with his ability to do math homework. The no-anecdote versions of the articles included descriptive text related to the topic of each intervention that was similar in length to the anecdotal stories. We hypothesized that participants would give higher ratings for the article that included an anecdote in terms of evidence strength, persuasiveness and likelihood of implementing the learning intervention. Additionally, we predicted that participants would be less likely to mention methodological flaws in their open-ended responses about the article that included an anecdote.

### Results

#### Evidence strength, persuasiveness and likelihood of implementing intervention

**Fig. 1:**
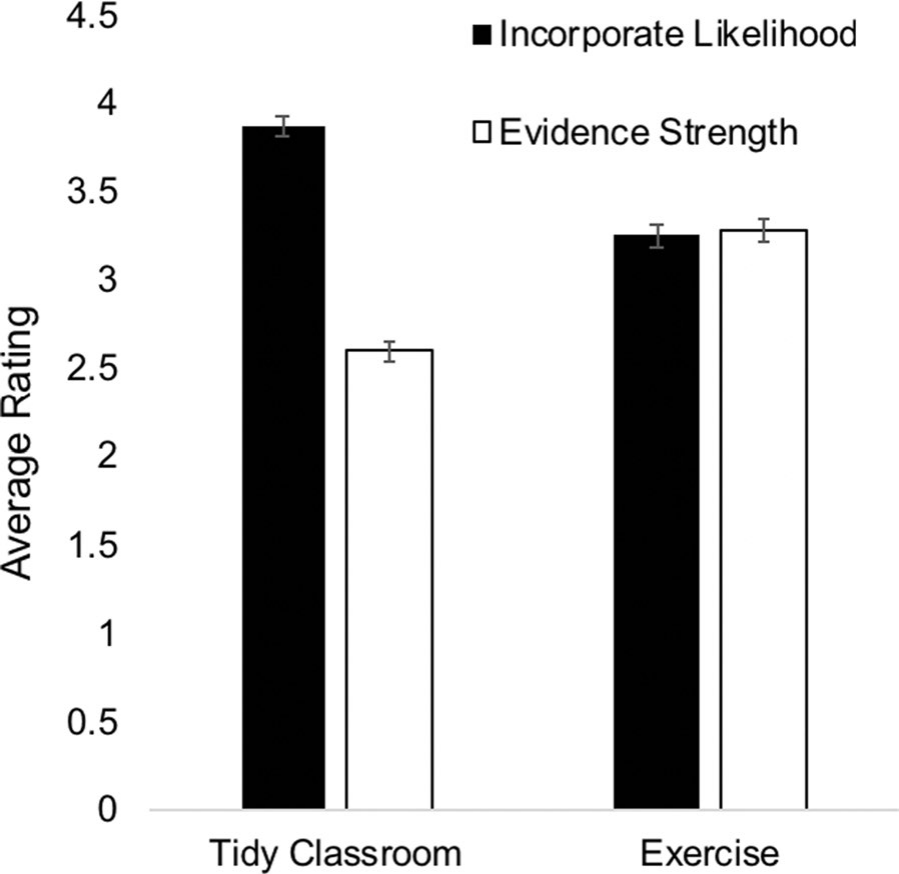
Means of ratings for evidence strength and incorporate likelihood as a function of intervention type (tidy classroom/exercise) for Experiment 1. Error bars represent within-subjects standard error of the mean (Cousineau, 2005)

We conducted a two-way ANOVA on ratings of evidence strength, persuasiveness and likelihood of implementing the intervention, with anecdote presence (present/absent) and intervention type (exercise/tidy classroom) as factors. Contrary to our hypothesis, there were no main effects of anecdote presence on participants’ overall ratings of evidence strength (*M* anecdote present = 2.99, *M* anecdote absent = 2.79), persuasiveness (*M* anecdote present = 3.29, *M* anecdote absent = 2.93) or likelihood of implementing the intervention (*M* anecdote present = 3.62, *M* anecdote absent = 3.60; all *F*’s < 2; see Table 1 for detailed descriptive statistics). Surprisingly, there were significant main effects of intervention type on ratings for evidence strength, *F*(1, 85) = 22.06, *p* < 0.001, η_p_^2^ = 0.09, persuasiveness, *F*(1, 85) = 18.64, *p* < 0.001, η_p_^2^ = 0.09, and likelihood of implementing the intervention, *F*(1, 85) = 13.35, *p* < 0.001, η_p_^2^ = 0.07, such that evidence strength and persuasiveness ratings were higher for the exercise (*M* evidence strength = 3.29; *M* persuasiveness = 3.49) than the tidy room intervention (*M* evidence strength = 2.60; *M* persuasiveness = 2.77). However, this effect was reversed for the likelihood of implementing the intervention rating, which was higher for the tidy room (*M* = 3.87) than the exercise intervention (*M* = 3.25; Fig. 1). We verified the different patterns of our dependent measures with a 2 × 2 repeated-measures ANOVA, with rating type (evidence strength/incorporate likelihood) and intervention type (exercise/tidy classroom) as within-subjects factors. There was a significant interaction between rating type and intervention type, *F*(1,86) = 64.03, *p* < 0.001, η_p_^2^ = 0.43. Post-hoc t-tests revealed that, whereas evidence strength (*M* = 3.29) and incorporate likelihood ratings (*M* = 3.25) were similar for the exercise intervention, *t*(86) = 0.27, *p* = 0.78, incorporate likelihood ratings were significantly higher (*M* = 3.87) than evidence strength ratings (*M* = 2.60) for the tidy classroom intervention, t(86) = 11.30, p < 0.001, *d* = 1.21.

**Table 1:** Descriptive statistics for article ratings and open-ended responses in Experiment 1

	Evidence Strength		Persuasiveness		Implement Likelihood	
	M	SD	M	SD	M	SD
Anecdote	2.99	1.23	3.29	1.21	3.62	1.18
No Anecdote	2.79	1.09	2.93	1.06	3.60	1.26
Exercise	3.29	1.10	3.49	0.96	3.25	1.18
Tidy Classroom	2.60	1.13	2.77	1.20	3.87	1.18

	Mention Study		Mention Personal Experience		Mention Participant Confound	
	M	SD	M	SD	M	SD
Exercise						
Anecdote	0.53	0.50	0.08	0.28	N/A	N/A
No Anecdote	0.47	0.51	0.18	0.39	N/A	N/A
Tidy Classroom						
Anecdote	0.61	0.49	0.47	0.51	0.47	0.51
No Anecdote	0.63	0.49	0.57	0.50	0.53	0.50

**Fig. 2:**
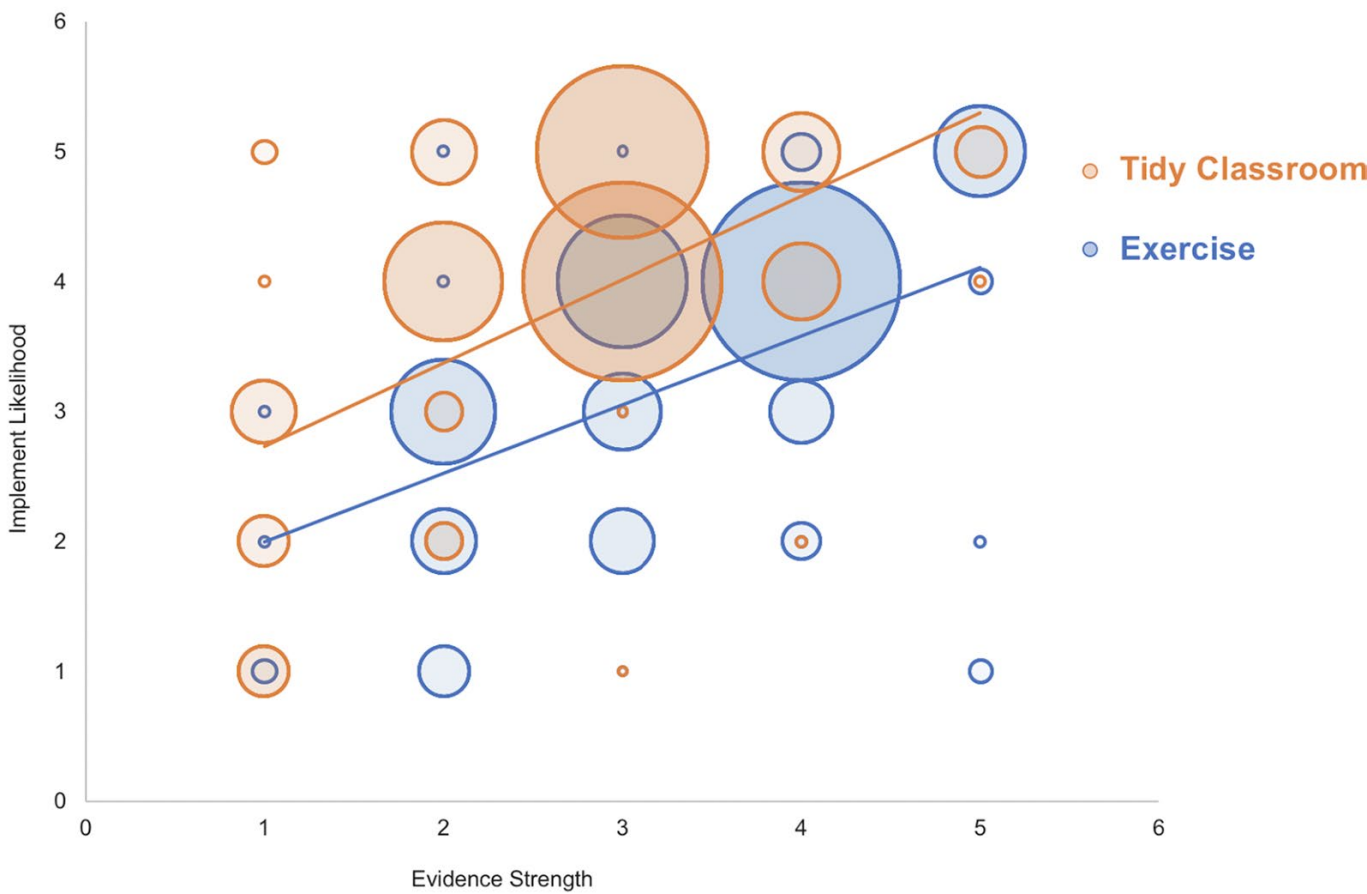
Correlations between ratings of evidence strength (x-axis) and likelihood of implementing the intervention (y-axis) for the exercise (blue) and tidy classroom (orange) interventions. The width of the bubbles represents the number of participants for each data point

We next asked to what extent the evidence strength and likelihood of implementing ratings were correlated for each intervention (Fig. 2). Although there were significant positive correlations between evidence strength and implement likelihood ratings for both the exercise (*r* = 0.45, *t*(85) = 4.69, *p* < 0.001) and tidy classroom interventions (*r* = 0.58, t(85) = 6.62, *p* < 0.001), participants showed a bias in which the likelihood of implementing the tidy classroom intervention was higher for a given level of evidence strength, as the higher orange trendline in Fig. 2 shows. For example, for evidence ratings of 3, most participants gave incorporate likelihood ratings of 4 or 5 for the tidy classroom intervention, whereas most participants gave incorporate likelihood ratings of 4 for the exercise intervention. Together, these data suggest that flawed scientific evidence was underweighed in decisions to implement the tidy classroom intervention.

#### Open-ended responses

To get a better understanding of these intervention effects, we analyzed participants’ open-ended explanations for evidence strength ratings and decisions to implement the intervention. Two raters (including one author, Y.Z.) coded the original responses independently; any discrepancies were resolved by a different author (A.M.). On average, the two raters achieved 85% agreement with a Cohen’s kappa value of 0.60. Specifically, we were interested in measuring how frequently participants mentioned certain aspects of the study (e.g., flaws in methods) as well as other factors that influenced their decisions, such as personal experiences and prior beliefs. For evidence rating explanations (responses to the question, “Please explain your reasoning: why did you rate the strength of evidence as weak/strong?”), we analyzed the number and types of methodological flaws that participants noticed (e.g., non-random assignment, other confounds). For the exercise article, only 11% of participants noticed that the study did not use random assignment, and only 2% noticed that an invalid measure (self-reporting) was used. In contrast, for the tidy classroom intervention, 51% of participants noticed that the study had a participant confound issue, and 14% noticed the priming confound. We conducted a one-way ANOVA to test for anecdote effects on mentions of the participant confound in the tidy classroom article; however, there was no significant difference between those who mentioned this flaw with the anecdote (*M* = 0.47) and the no anecdote (*M* = 0.53) version of the article (*F* < 0.5). Because so few participants noticed the other specific flaws in the articles, we did not statistically analyze the effect of anecdotes on those responses.

**Fig. 3:**
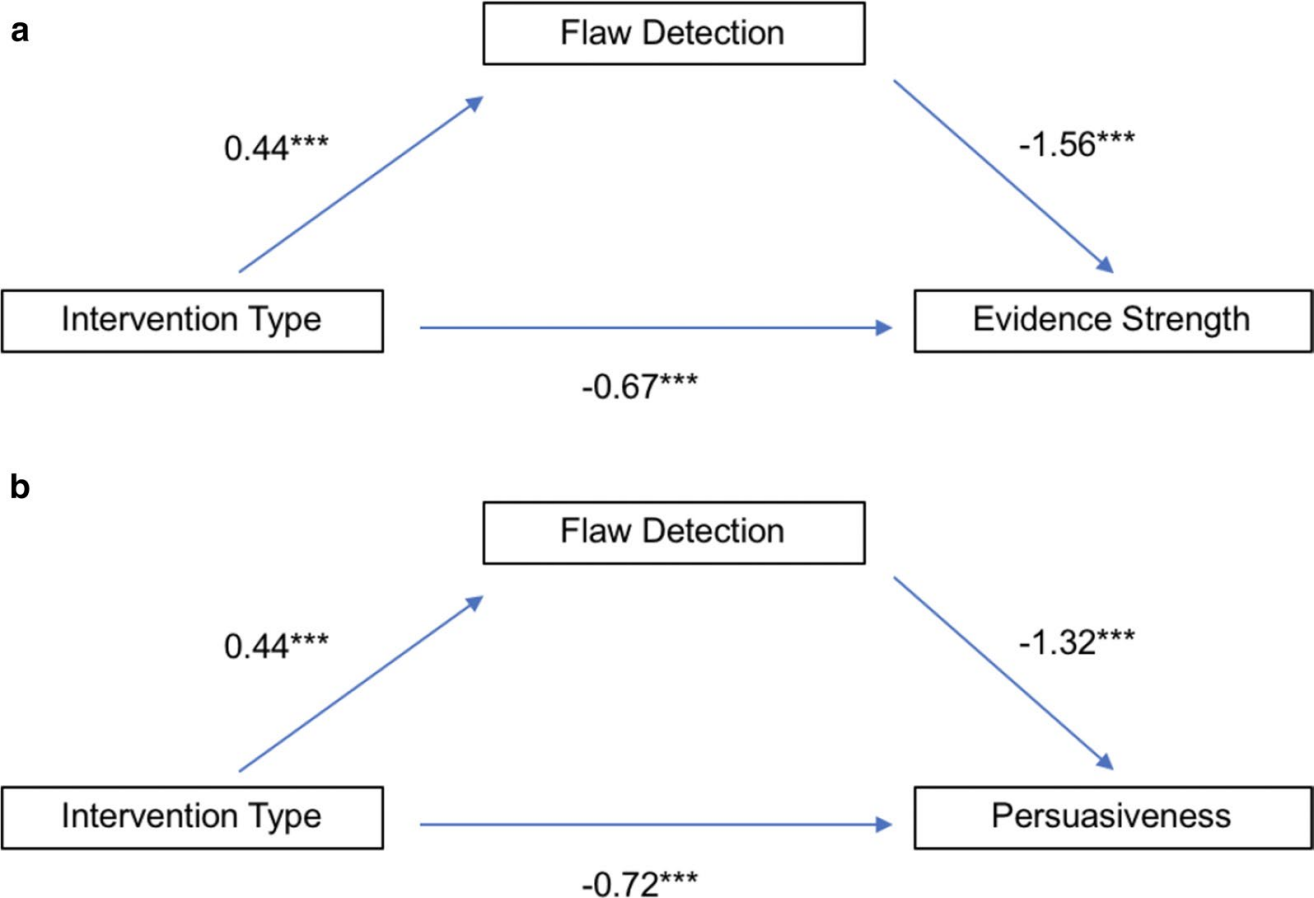
Mediation analysis showing direct and indirect effects (via detection rates of study flaws) of intervention type on evidence strength ratings (**a**) and on persuasiveness ratings (**b**)

Together, these findings suggest that participants were more likely to notice methodological flaws in the tidy classroom than the exercise intervention article, perhaps because the flaws were more obvious. To test whether intervention effects on evidence strength and persuasiveness ratings were indirectly impacted by the salience of the flaws in the studies, we conducted mediation analyses in which intervention type was the independent variable, mentions of study flaws was the mediator, and evidence strength or persuasiveness rating was the dependent variable. This analysis revealed that the effects of intervention type on both evidence strength and persuasiveness ratings were significantly mediated by study flaw detection (Fig. 3; evidence strength ACME = − 0.68, 95% C.I. (− 0.91, − 0.47); persuasiveness ACME = − 0.58, 95% C.I. (− 0.79, − 0.39).

For explanations about decisions to implement the intervention (e.g., responses to the question, “Please explain your reasoning: why would you choose/not choose to make sure that your classroom is tidy during lessons and exams?”), we first coded whether participants mentioned the study itself as the basis for their decision (e.g., “The evidence shows that exercising while learning is effective, therefore, I would choose to incorporate it in hopes of my students being able to learn better,”; “There is bias in the study so it's not conclusive,”). If participants’ explanations mentioned the study, either positively or negatively, their responses were coded as a 1; otherwise, they were coded as a 0. Additionally, if participants mentioned their personal experience or prior beliefs (e.g., “I am a very clean person and feel that I would keep my classroom clean regardless…”), their response was coded as a 1; otherwise, it was coded as a 0. Note that these ‘mention study’ and ‘mention personal experience’ codes were not mutually exclusive. We conducted a two-way ANOVA (with anecdote presence and intervention type as factors) on ‘mention study’ and ‘mention personal experience’ rates for explanations for decisions to implement the interventions. There was a trend for a main effect of intervention type on the ‘mention study’ rate, *F*(1, 85) = 3.44, *p* = 0.07, η^2^_p_ = 0.01, such that participants were more likely to mention the study as a decision factor for the tidy classroom (*M* = 0.62) than the exercise intervention (*M* = 0.51). There was also a significant main effect of intervention type on the ‘mention personal experience’ rate, *F*(1, 85) = 39.37, *p* < 0.001, η^2^_p_ = 0.17, such that participants were also more likely to mention personal experience or prior beliefs as the basis for their decision to implement the tidy classroom (*M* = 0.53) than the exercise intervention (*M* = 0.13).

Thus, although participants were more likely to notice methodological flaws in the description of the tidy classroom intervention study, at the same time, participants were more likely to cite their own personal experiences, opinions and beliefs as reasons for adopting the tidy classroom than the exercise intervention. We thus speculated that the two interventions may have differed in their baseline plausibility. To test the possibility that the tidy classroom intervention was more plausible as a potential educational intervention, we conducted a post-hoc follow-up survey on Amazon’s Mechanical Turk with a separate group of participants (*N* = 94). Participants responded to three questions about each intervention (presentation order was randomized) using a Likert scale: 1) how much experience they themselves have had with the intervention (e.g., exercising while studying or straightening up before studying; 1 = never, 5 = always), 2) the extent to which they believe that the intervention is effective for improving learning (1 = none at all, 5 = a great deal), and 3) how likely it is that a hypothetical research study would find that the intervention leads to learning improvements in a group of people (1 = extremely unlikely, 5 = extremely likely). Participants gave significantly higher ratings for the tidy classroom than the exercise intervention for all three measures (personal experience: *M* tidy classroom = 3.5, *M* exercise = 2.2, *t*(93) = 9.35, *p* < 0.001, *d* = 1.08; belief about effectiveness: *M* tidy classroom = 3.9, *M* exercise = 2.4, t(93) = 10.02, p < 0.001, *d* = 1.24; prediction for positive study outcome: *M* tidy classroom = 4.2, *M* exercise = 3.0, *t*(93) = 8.19, *p* < 0.001, *d* = 1.09; see Table 2 for detailed descriptive statistics). Thus, baseline differences in personal experience with and plausibility of the interventions could explain why participants in Experiment 1 tended to implement the tidy classroom intervention, despite acknowledging that the evidence in favor of the technique was weak.

**Table 2:** Descriptive statistics for baseline beliefs about interventions

	Personal Experience		Belief about Effectiveness		Prediction for Possible Study Outcome	
	M	SD	M	SD	M	SD
Exercise	2.21	1.25	2.44	1.24	3.00	1.27
Tidy Classroom	3.47	1.09	3.86	1.05	4.18	.87

Our findings are consistent with previous work showing that personal experiences and prior beliefs are weighted heavily in decisions (Garcia-Retamero et al., 2009). In particular, people struggle to update their beliefs when presented with new compelling evidence that conflicts with their initial belief (Ecker et al., 2014; Lewandowsky et al., 2012), and this problem persists even among trained scientists (Koehler, 1993). In fact, the opposite may occur; people may cling to their initial beliefs more strongly when faced with conflicting evidence, perhaps by discounting the data (Lord et al., 1979; Trevors et al., 2016; though see Wood and Porter (2019), who find little evidence in support of this so-called “backfire effect”). Here we find that even when people do not discount the evidence (i.e., they acknowledge that the evidence in support of the tidy classroom intervention is weak), their prior belief (e.g., that having a tidy room is helpful for learning) may override their evidence evaluations when making decisions. For example, when explaining why they would choose to implement the tidy classroom intervention in a hypothetical classroom, one participant responded: “Although the evidence in this article was not very strong, it is easy to have students keep an organized desk and have a tidy room with less distractions, so I would probably incorporate this into my teaching.” Another said, “I would choose to make my classroom tidy because, regardless of the convincingness of this article, performing better in a tidy room seems like a logical conclusion that I am willing to support.” Thus, we observed a dissociation between participants’ ability to *acknowledge* flawed evidence and their ability to *use* flawed evidence appropriately when making decisions.

Contrary to our prediction, we did not find that the presence of anecdotes affected either evaluations of or decisions based on low-quality research in Experiment 1. The lack of an anecdote effect was surprising, given that a substantial amount of research (reviewed above) has established the relative importance of anecdotes in both evidence evaluation and decision-making. In particular, we were unable to replicate previous findings that the presence of anecdotes reduced evidence evaluation and scientific reasoning (Rodriguez, Rhodes, et al., 2016). However, further inspection revealed that there were substantial differences between the current study and the Rodriguez, Rhodes, et al., (2016) study. First, whereas we included anecdote presence as a within-subjects factor, anecdote presence was completely between-subjects in the Rodriguez, Rhodes, et al., (2016) study. Additionally, whereas participants only rated a single article in the presence of an anecdote in the current study, participants in the Rodriguez, Rhodes, et al., (2016) study rated 8 articles. Thus, it is possible that anecdotes may only influence evaluations of evidence quality under certain testing conditions. Further work is also needed to determine whether anecdotes might show a stronger influence on evidence-based decision-making when anecdote presence is a between-subjects factor, when participants are given the opportunity to evaluate a greater number of research studies, and/or when the topic of research is more neutral in regards to participants’ prior beliefs.

There were some other limitations to Experiment 1; first, given that we found baseline differences in prior beliefs about plausibility between the tidy classroom and exercise interventions in our follow-up survey on Mechanical Turk, we wanted to test whether the intervention effect on incorporate likelihood ratings was in fact mediated by participants’ prior beliefs about the plausibility of these interventions. However, because we collected plausibility ratings about the interventions from a separate sample, we were unable to conduct a mediation analysis for data in Experiment 1. Thus, in Experiment 2, we conducted an experiment in which we first asked participants about their beliefs about the plausibility of these interventions before having them evaluate the flawed studies about the interventions. Second, because we did not anticipate that incorporate likelihood ratings would differ between the two learning interventions, we did not specifically test a range of interventions that systematically varied in their plausibility. It is thus unclear whether our results are generalizable to other contexts beyond the tidy classroom and exercise interventions. In Experiment 2, we additionally tested whether participants would be more likely to incorporate other learning interventions considered to be highly plausible compared to less plausible interventions. Finally, although the effect of intervention type on evidence strength ratings had a sufficient level of power (99% for alpha = 0.05), the intervention type effect on incorporate likelihood ratings was underpowered in Experiment 1 (71% for alpha = 0.05). Thus, we wanted to replicate our findings with a larger sample size to achieve a sufficient level of power.

To address these limitations, we conducted a second experiment with two parts. In an initial pretest, our goal was to find two additional interventions that varied strongly in their baseline plausibility to extend our findings from Experiment 1 to other contexts. In Experiment 1, we found that the exercise intervention was perceived as implausible for two reasons: participants did not believe it would be effective for improving learning, and they thought it was impractical (e.g., it would be distracting to exercise while studying and/or difficult to implement logistically in a classroom setting). Thus, in the pretest for Experiment 2 we tested three possible learning interventions that we thought might be perceived as both ineffective and impractical: napping at school, singing learned material, and doodling while learning. For the high plausible intervention, we chose virtual/augmented reality because we have previously found that people have strong beliefs about its effectiveness as a learning intervention (unpublished data).

The participants for this pretest were 100 paid participants recruited from Prolific (http://prolific.co). Participants were paid $9.50 per hour. All participants consented to participate in the study, which was approved by the University of Michigan Institutional Review Board. Participants responded to a brief online survey on Qualtrics that asked about their prior beliefs about four classroom learning interventions that might improve retention of newly learned material: virtual/augmented reality, napping, singing, and doodling (i.e., open-ended drawing). For each intervention, participants used a Likert scale to respond 1) how effective they thought the intervention would be compared to a control condition (e.g., “Do you think learning is better when people use virtual or augmented reality technology compared to reading slides on a computer?”; 1 = none at all, 5 = a great deal); 2) whether they personally have tried the intervention themselves (e.g., “Have you ever tried using virtual or augmented reality technology to learn about something?”; 1 = never, 5 = always); and 3) how practical they believed the intervention would be in a classroom setting (e.g., “How practical do you think it would be to have students use virtual or augmented reality technology to learn in a classroom setting?”; 1 = none at all, 5 = a great deal). Participants were asked about each of the four learning interventions in random order. Based on previous testing, we hypothesized that participants would rate the virtual reality intervention as highly effective for learning.

We first conducted a one-way repeated measures ANOVA on intervention effectiveness ratings, with intervention type as a within-subjects factor. There was a significant main effect of intervention type, F(3,297) = 9.11, p < 0.001, *η*_*p*_^*2*^ = 0.08. As predicted, the virtual reality intervention had the highest average effectiveness rating (see Table 3 for a summary of descriptive statistics for all intervention and question types). Post-hoc t-tests revealed that effectiveness ratings for the virtual reality intervention (*M* = 3.39) were significantly higher than the napping intervention (*M* = 2.63), *t*(99) = 4.76, *p* < 0.001, *d* = 0.48, but did not differ significantly from either the singing or the doodling interventions (all *t*’s < 2). We next conducted a similar ANOVA on practicality ratings and found a significant main effect of intervention type, *F*(3,297) = 32.86, *p* < 0.001, *η*_*p*_^*2*^ = 0.25. The virtual reality and doodling interventions had the highest average practicality ratings (3.18 and 3.19, respectively). Post-hoc t-tests revealed that practicality ratings for the virtual reality intervention (*M* = 3.18) were significantly higher than both the napping intervention (*M* = 2.05), *t*(99) = 8.17, *p* < 0.001, *d* = 0.82, and the singing intervention (*M* = 2.60), *t*(99) = 4.59, *p* < 0.001, *d* = 0.46; similarly, practicality ratings for the doodling intervention were significantly higher than both the napping intervention, *t*(99) = 7.51, *p* < 0.001, *d* = 0.75, and the singing intervention, *t*(99) = 4.40, *p* < 0.001, *d* = 0.44. Finally, we conducted a similar ANOVA on personal experience ratings; there was a significant main effect of intervention type, *F*(3,297) = 18.61, *p* < 0.001, *η*_*p*_^*2*^ = 0.16, such that ratings for the doodling intervention (*M* = 2.83) were significantly higher than all other intervention types (all *t*’s > 4.5). However, since we were mainly interested in differences in prior beliefs about the plausibility of these interventions, we only considered the effectiveness and practicality effects from this experiment.

**Table 3:** Descriptive statistics for baseline beliefs about interventions for pretest to Experiment 2

	Effectiveness		Practicality		Personal Experience	
	M	SD	M	SD	M	SD
Virtual Reality	3.39	1.04	3.18	0.90	1.95	1.06
Napping	2.63	1.14	2.05	1.17	1.91	1.03
Singing	3.18	1.22	2.60	1.11	2.13	1.06
Doodling	3.15	1.22	3.19	1.13	2.83	1.24

Because the differences in effectiveness and practicality were largest between the virtual reality and napping interventions, we chose to use these interventions in our replication of Experiment 1. The virtual reality intervention was rated as the more effective and more practical intervention; thus, we chose to use it as a second example of a *high plausible* learning intervention, similar to the tidy classroom intervention from Experiment 1. Because the napping intervention was rated as significantly less effective and less practical than the virtual reality intervention, we chose to include it as a second example of a *low plausible* learning intervention, similar to the exercise intervention from Experiment 1.

## Experiment 2

The goals of Experiment 2 goals were threefold: first, we wanted to test the hypothesis that the effect of intervention type on incorporate likelihood ratings from Experiment 1 was mediated by prior beliefs about the plausibility of the interventions. Because we did not collect intervention plausibility ratings and article evaluation ratings from the same sample in Experiment 1, in Experiment 2, we ran an experiment in which the same participants rated both their prior beliefs about the interventions and their evaluations and decisions about the articles. Second, we wanted to test whether our findings from Experiment 1 would extend to other learning interventions beyond the tidy classroom and exercise interventions. In the pretest to Experiment 2, we tested a separate group of participants’ prior beliefs about the plausibility (i.e., effectiveness and practicality) of four possible learning interventions in order to find an additional *high plausible* intervention (similar to the tidy classroom intervention) and *low plausible* intervention (similar to the exercise intervention). Based on our results from the pretest, these new interventions included a virtual/augmented reality intervention for the *high plausible* condition and a napping intervention for the *low plausible* condition. Finally, we wanted to replicate our findings from Experiment 1 with a larger sample size, given that the intervention effect on incorporate likelihood ratings was underpowered in Experiment 1. Using the pwr package (v. 1.2–2; Champely, 2018) in R, we determined that we would need 107 participants to achieve a similar effect size as in Experiment 1 with 80% power at *ɑ* = 0.05, which we rounded up to 110 participants.

### Method

#### Participants

The participants for this study were 110 paid participants recruited from Prolific (http://prolific.co). Participants were paid $9.50 per hour. All participants consented to participate in the study, which was approved by the University of Michigan Institutional Review Board.

#### Procedure

Participants completed an online survey using Qualtrics. Similar to the pretest to Experiment 2, participants first responded to a prior belief pretest consisting of a set of three questions about the plausibility of four classroom learning interventions: having a tidy classroom while learning, exercising while learning, virtual/augmented reality while learning, and napping after learning. The question and response formats were identical to those used in the pretest to Experiment 2, and the interventions were presented in random order. Next, participants read four fictitious articles (again presented in random order) that were similar in format to the non-anecdote articles used in Experiment 1. Each article featured one of the classroom interventions asked about in the prior belief pretest described above (see "Appendix 2" for examples of the virtual reality and napping articles). Participants responded to the same questions asked in Experiment 1: they rated each article in terms of its evidence strength, persuasiveness, and the likelihood that they would implement the intervention in a hypothetical classroom, and they explained their reasoning for evidence strength and incorporate likelihood ratings in an open-ended way.

#### Materials

The tidy classroom and exercise intervention articles were identical to the non-anecdote versions of the articles from Experiment 1. For the virtual/augmented reality article, the description of the study was based off of a study done by Parong and Mayer (2018), though we modified the original results so that participants in the virtual reality group outperformed the Powerpoint group. The two major methodological flaws we planted were that participants were assigned to groups based on skill level (non-random assignment) and there were an uneven number of participants in each group (i.e., 10 in one group versus 50 in the other group). For the napping article, the description of the study was based off of a study done by Cabral et al. (2018) and modified to include two major methodological flaws: participants could choose whether to nap or not (non-random assignment), and participants self-reported how much they felt they remembered learning rather than completing a test (invalid measure).

We hypothesized that participants would be more likely to incorporate the *high plausible* interventions than the *low plausible* interventions; furthermore, we expected to observe a dissociation between evidence strength ratings and decisions to incorporate the interventions, such that incorporate likelihood ratings would be higher than evidence strength ratings for the *high plausible* interventions (replicating findings from Experiment 1). Additionally, we hypothesized that prior beliefs about effectiveness and practicality would mediate any effects of intervention plausibility on incorporate likelihood ratings.

### Results

#### Prior belief ratings

Table 4 presents a summary of descriptive statistics for participants’ prior beliefs about the effectiveness, personal experience with, and practicality of the four learning interventions. To confirm that the selected *high plausible* interventions were in fact perceived as more plausible than the selected *low plausible* interventions, we used paired t-tests to compare averages of effectiveness and practicality ratings for the virtual/augmented reality and tidy classroom interventions (*high plausible*) to averages of effectiveness and practicality ratings for the napping and exercise interventions (*low plausible*). The *high plausible* interventions were rated as significantly more effective (*M* high plausible = 3.58; *M* low plausible = 2.58, *t*(109) = 9.04, *p* < 0.001, *d* = 0.90) and more practical (*M* high plausible = 3. 34, *M* low plausible = 2.24, *t*(109) = 11.44, *p* < 0.001, *d* = 1.09.

**Table 4:** Descriptive statistics for prior belief ratings for interventions in Experiment 2

	Effectiveness		Practicality		Personal Experience	
	M	SD	M	SD	M	SD
Virtual Reality	3.28	1.08	3.15	1.10	1.76	1.11
Tidy Classroom	3.87	1.16	3.70	1.09	3.18	1.17
Napping	2.95	1.27	2.34	1.21	2.09	0.96
Exercise	2.21	1.15	2.15	1.08	1.74	0.90

#### Evidence strength, persuasiveness and likelihood of implementing intervention ratings

We conducted a repeated-measures ANOVA to ask whether article ratings differed by plausibility of the intervention (*high*/*low* plausible, within-subjects factor; see Table 5 for a summary of descriptive statistics for ratings). Ratings were significantly higher for the *high plausible* than *low plausible* interventions for evidence strength (*M* high plausible = 3.56, *M* low plausible = 3.21, *F*(1,109) = 21.36, *p* < 0.001, *η*_*p*_^*2*^ = 0.16), persuasiveness (*M* high plausible = 3.66, *M* low plausible = 3.18, *F*(1,109) = 45.20, *p* < 0.001, *η*_*p*_^*2*^ = 0.29) and likelihood of incorporating the intervention (*M* high plausible = 4.09, *M* low plausible = 2.79, *F*(1,109) = 118.10, *p* < 0.001, *η*_*p*_^*2*^ = 0.52). We thus replicated the effect of intervention type on incorporate likelihood ratings from Experiment 1. In contrast to Experiment 1, however, we found that evidence strength ratings were higher for the *high plausible* than *low plausible* interventions in Experiment 2. We address possible explanations for this discrepancy later on in our analysis of open-ended explanations of evidence strength ratings (see Table 5 for a summary of descriptive statistics of open-ended responses).

**Table 5:** Descriptive statistics for article ratings and open-ended responses for Experiment 2

	Evidence Strength		Persuasiveness		Implement Likelihood	
	M	SD	M	SD	M	SD
Low Plausible	3.21	1.03	3.18	1.05	2.79	1.36
Exercise	3.33	1.02	3.32	1.01	3.11	1.29
Napping	3.10	1.03	3.04	1.07	2.47	1.37
High Plausible	3.56	1.09	3.66	1.08	4.09	1.12
Tidy Classroom	3.79	1.02	3.75	1.08	4.39	0.88
Virtual Reality	3.34	1.10	3.58	1.07	3.79	1.26

	Mention Study		Mention Personal Experience/Beliefs			
	M	SD	M	SD		
Low Plausible	0.24	0.35	0.24	0.32		
Exercise	0.26	0.44	0.22	0.41		
Napping	0.21	0.41	0.25	0.44		
High Plausible	0.20	0.34	0.58	0.36		
Tidy Classroom	0.22	0.41	0.63	0.49		
Virtual Reality	0.17	0.38	0.53	0.50		

**Fig. 4:**
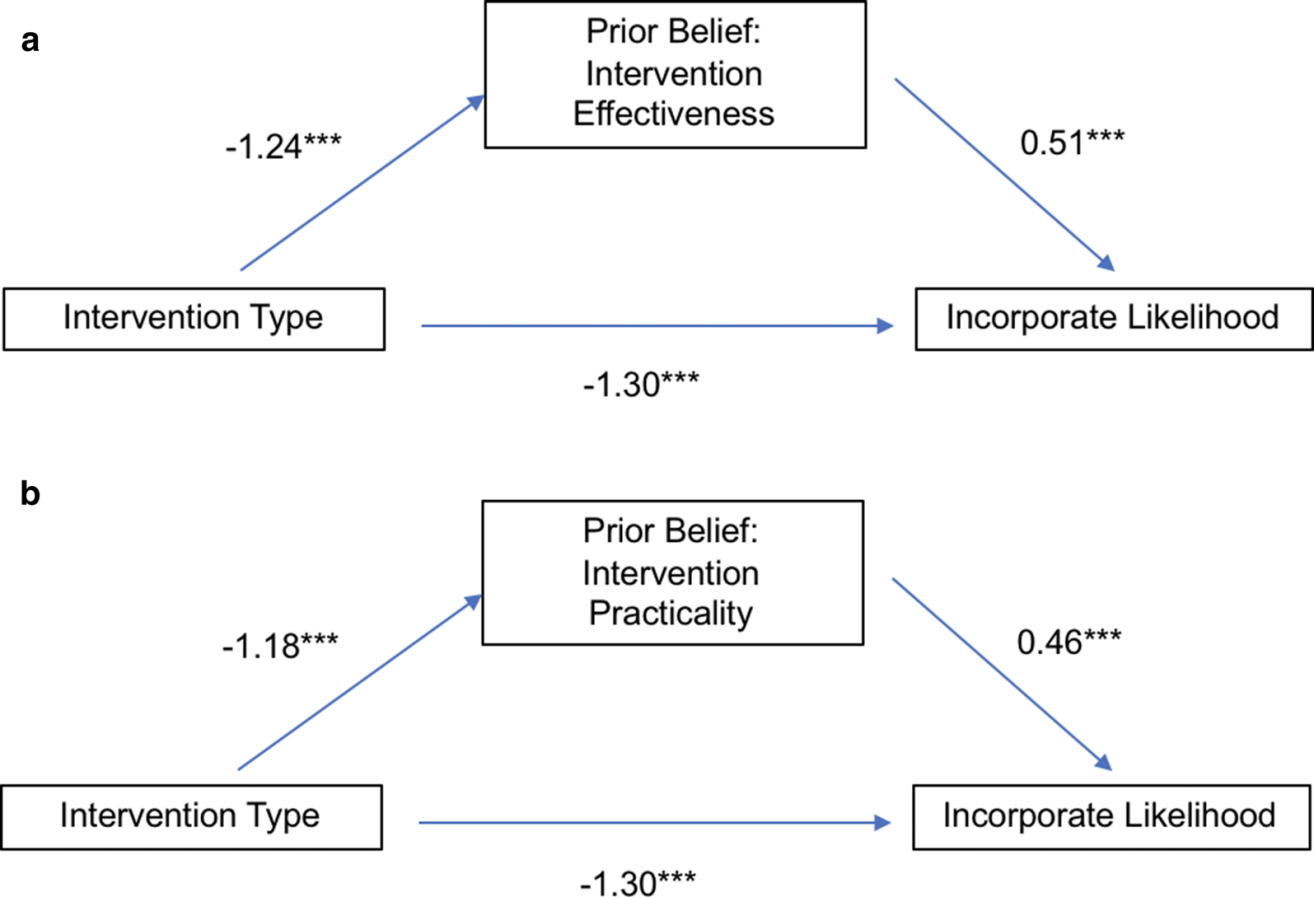
Mediation analysis showing direct and indirect effects of intervention type as mediated by prior beliefs about intervention effectiveness (**a**) and intervention practicality (**b**) on incorporate likelihood ratings

We next tested our hypothesis that the effect of intervention plausibility on incorporate likelihood ratings was mediated by participants’ prior beliefs about the interventions; specifically, we conducted two separate mediation analyses to test the indirect effects of beliefs about intervention effectiveness and practicality. Thus, the independent variable was intervention plausibility (*high*/*low*), the mediators were intervention effectiveness and practicality (averaged across the two *high plausible* and two *low plausible* interventions), and the dependent variable was incorporate likelihood ratings (averaged across the two *high plausible* and *low plausible* interventions). As shown in Fig. 4, prior beliefs about both intervention effectiveness and practicality significantly mediated the effect of intervention plausibility on incorporate likelihood ratings (effectiveness: ACME = − 0.64, 95% C.I. = [ − 0.84, − 0.43], *p* < 0.001; practicality: ACME = − 0.54, 95% C.I. = [ − 0.72, − 0.37], *p* < 0.001).

**Fig. 5:**
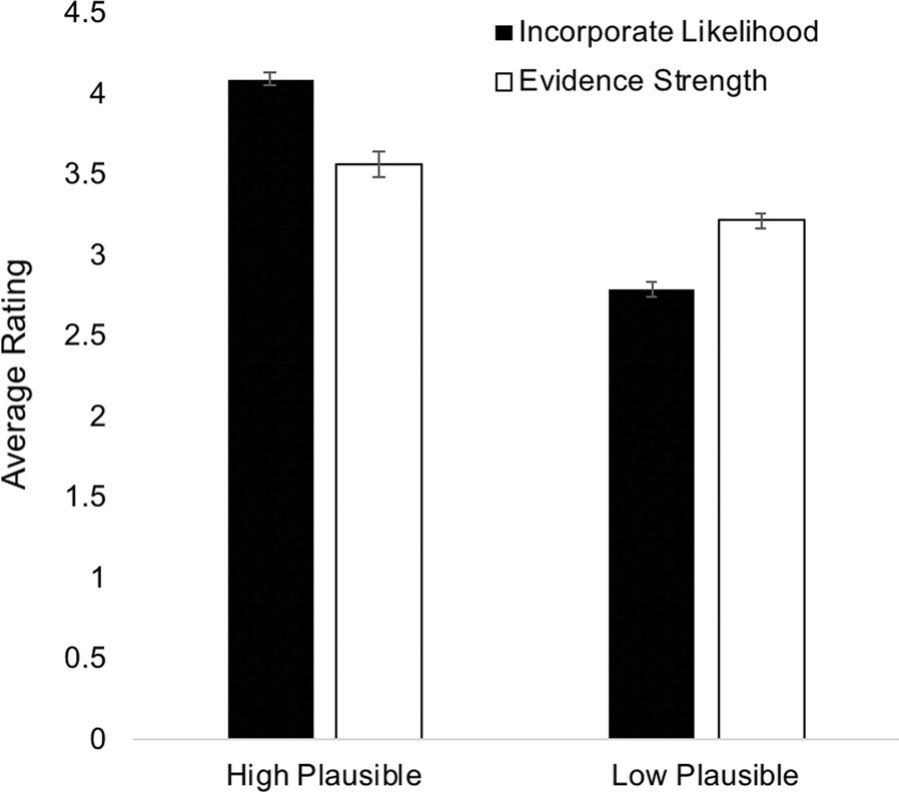
Means of ratings for evidence strength and incorporate likelihood as a function of intervention plausibility for Experiment 2. Error bars represent within-subjects standard error of the mean (Cousineau, 2005)

We next tested relationships between evidence strength and incorporate likelihood ratings as a function of intervention plausibility. First, we conducted a 2 × 2 repeated-measures ANOVA with intervention plausibility (*high*/*low*) and rating type (evidence strength/incorporate likelihood) as within-subjects factors. There was a significant interaction between intervention plausibility and rating type, *F*(1,109) = 25.54, *p* < 0.001, *η*_*p*_^*2*^ = 0.41. As shown in Fig. 5, incorporate likelihood ratings were significantly lower than evidence strength ratings for the *low plausible* interventions, *t*(109) = 4.88, *p* < 0.001, *d* = 0.46, whereas incorporate likelihood ratings were significantly higher than evidence strength ratings for the *high plausible* interventions, *t*(109) = 7.19, *p* < 0.001, *d* = 0.69. Thus, similar to Experiment 1, participants were more likely to incorporate *high plausible* learning interventions given their evidence strength ratings. In contrast to Experiment 1, we found that participants were less likely to incorporate *low plausible* learning interventions given their evidence strength ratings.

**Fig. 6:**
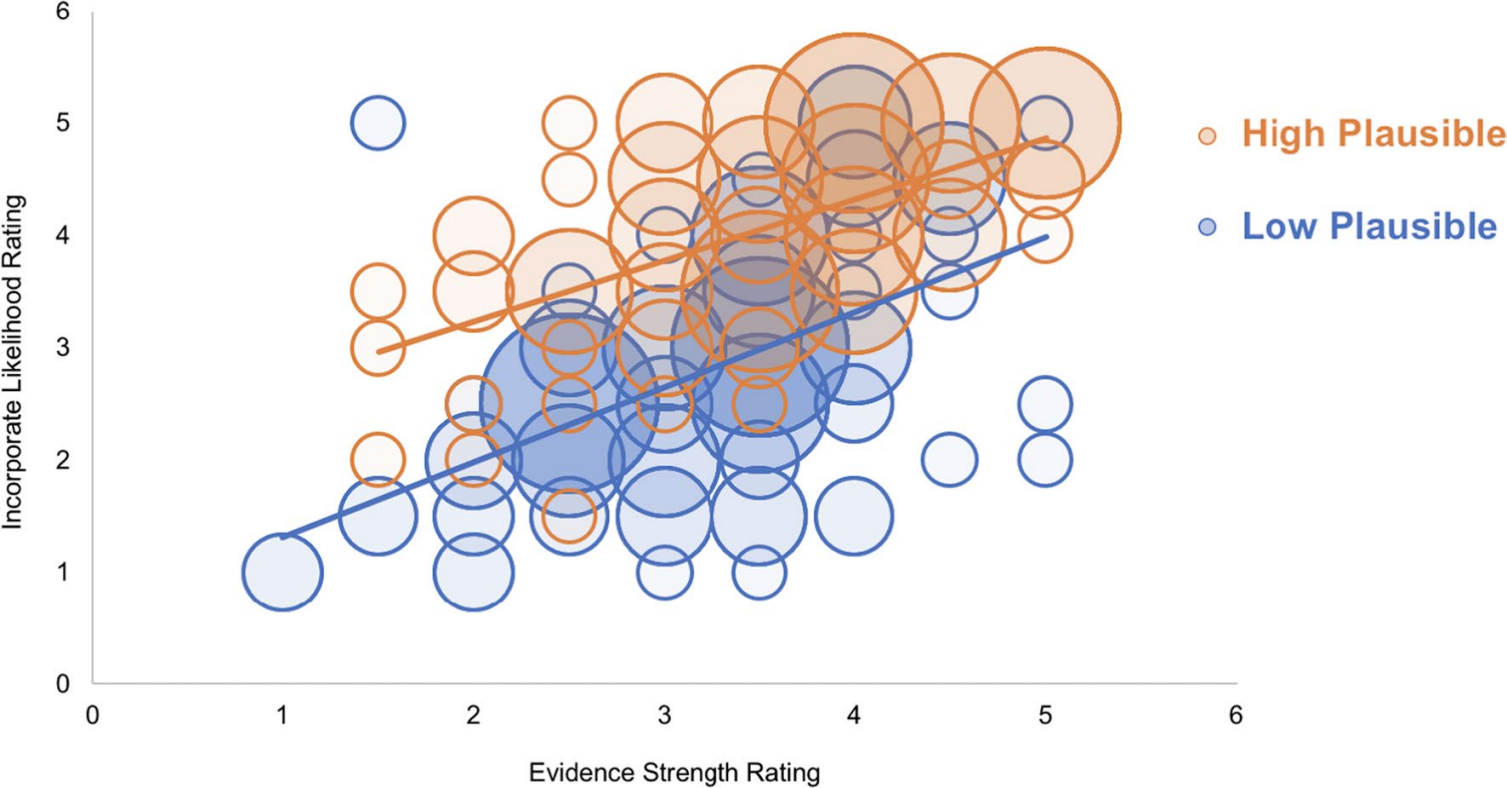
Correlations between evidence strength and incorporate likelihood ratings as a function of intervention plausibility (*high plausible* = orange, *low plausible* = blue) for Experiment 2. Size of bubbles reflects the number of participants for each data point

We again confirmed that correlations between evidence strength and incorporate likelihood ratings differed as a function of intervention plausibility. As shown in Fig. 6, we found significant positive correlations for both *low plausible* (*r* = 0.53, *t*(108) = 6.55, *p* < 0.001) and *high plausible* interventions (*r* = 0.58, *t*(108) = 7.38, *p* < 0.001). However, as in Experiment 1, for a given evidence strength rating, incorporate likelihood ratings were higher for the *high plausible* than *low plausible* interventions, as indicated by the higher overall trendline for the *high plausible* interventions. Together, these findings suggest that participants underweighed evidence strength as a factor in their decisions to implement learning interventions; rather, the plausibility of the learning interventions was the stronger predictor.

#### Open-ended responses

Similar to Experiment 1, we coded participants’ open-ended explanations for their evidence strength and incorporate likelihood ratings (see Table 5 for a summary of descriptive statistics for open-ended responses). Two raters (including one author, Y.Z.) coded the original responses independently; any discrepancies were resolved by a different author (A.M.). On average, the two raters achieved 85.6% agreement with a Cohen’s kappa value of 0.53 (lowest kappa value = 0.37, for explanations for incorporating interventions). We first analyzed the number and types of methodological flaws that participants noticed for each intervention. Although we did not specifically manipulate the sample size as a methodological flaw, we nevertheless noticed that many participants cited sample size (either as being too low or sufficiently high) as part of their explanations for their evidence strength ratings; thus, we also analyzed the number of participants who mentioned sample size in their explanations.

We first analyzed evidence strength explanations for the *low plausible* interventions (exercise and napping). For the exercise intervention, 5% of participants noticed that groups were not randomly assigned, 2% of participants noticed that an invalid measure was used, and 27% of participants mentioned a small sample size (*N* = 60). For the napping intervention, 3% of participants noticed that groups were not randomly assigned, 15% of participants noticed that an invalid measure was used, and 13% of participants noted that the sample size was low (*N* = 125). We next analyzed mentions of methodological flaws for the *high plausible* interventions (tidy classroom and virtual reality). For the tidy classroom intervention, 10% of participants noticed the participant confound, 5% of participants noticed the priming confound, and 7% of participants cited a low sample size (*N* = 225). Finally, for the virtual reality intervention, 11% of participants noticed that the two groups had uneven numbers, 11% of participants noticed that groups were not randomly assigned, and 34% of participants cited a small sample size (*N* = 60). Given that evidence strength ratings were significantly higher for *high* than *low* plausible interventions, we ran a repeated-measures ANOVA on the total number of flaws detected (excluding sample size) with intervention plausibility (high/low) as a within-subjects factor to test whether participants were more likely to mention study flaws for *low* than *high* plausible interventions. However, flaw detection rates did not differ significantly between *high plausible* (*M* = 0.18) and *low plausible* interventions (*M* = 0.13), *F*(1,109) = 3.51, *p* = 0.064. We also ran a similar analysis including sample size detection in the total flaw number; however, there was once again no significant effect of intervention plausibility (high plausible *M* = 0.39; low plausible *M* = 0.33, *F*(1, 109) = 2.56, *p* = 0.11). Thus, evidence strength ratings were higher for *high plausible* than *low plausible* interventions despite the fact that there were no differences in flaw detection rates for the two types of interventions. This is in contrast to our findings in Experiment 1, in which participants were more likely to notice flaws in the more plausible intervention (tidy classroom), and flaw detection rates significantly mediated the effect of intervention type on evidence strength ratings.

**Fig. 7:**
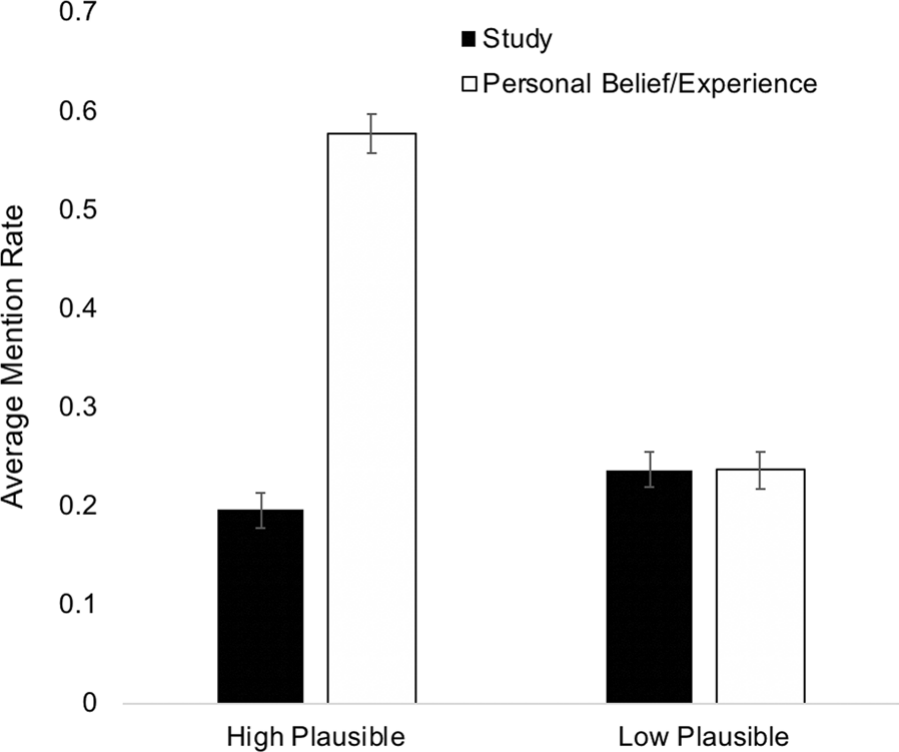
Rates of mentioning the study and personal experiences/beliefs for explanations for incorporate likelihood ratings as a function of intervention plausibility (high/low) for Experiment 2. Error bars represent within-subjects standard error of the mean (Cousineau, 2005)

We also coded and analyzed participants’ explanations for their incorporate likelihood ratings. Specifically, we coded whether participants mentioned the study and/or their personal beliefs/experience as reasons for incorporating the learning intervention in a hypothetical classroom, using the same criteria and procedure as in Experiment 1. We then ran a repeated-measures ANOVA on ‘mention study’ rates and ‘mention personal belief/experience rates’ with intervention plausibility (high/low) as a within-subjects factor. Although participants were equally likely to mention the study as a basis for their decision for *high plausible* (*M* = 0.20) and *low plausible* interventions (*M* = 0.23, *F*(1,109) = 1.38, *p* = 0.24), they were significantly more likely to mention personal beliefs/experience as the basis for their decision for *high plausible* (*M* = 0.58) than *low plausible* interventions (*M* = 0.24, *F*(1,109) = 79.78, *p* < 0.001, *η*_*p*_^*2*^ = 0.42), replicating our finding from Experiment 1. We additionally ran a repeated-measures ANOVA to directly compare the explanation types (mention study/mention personal beliefs and experience) as a function of intervention plausibility (high/low) and found a significant interaction, such that participants were more likely to cite personal beliefs/experiences than the study only for the *high plausible* interventions, *F*(1,109) = 49.99, *p* < 0.001, *η*_*p*_^*2*^ = 0.31 (Fig. 7). Thus, similar to Experiment 1, participants were more likely to cite personal beliefs/experience as their reason for implementing *high* than *low plausible* interventions; in contrast to Experiment 1 participants were more likely to mention personal beliefs/experience than the study as a reason for their decision only for the *high plausible* interventions.

### Discussion

Here, we examined how people simultaneously weigh poor quality evidence (i.e., bad science) in the context of anecdotal evidence and belief-consistent and belief-inconsistent topics when making evidence-based decisions about educational studies described in the popular press. In Experiment 1, we tested whether the presence of an anecdote would inflate the perceived quality of evidence and increase the likelihood that participants would act on flawed studies about two learning interventions: taking an exam in a tidy classroom and exercising while learning. Although including an anecdote did not affect participants’ evidence evaluations or decision-making, we found a dissociation between evidence evaluation and decision-making: participants were more likely to adopt the intervention that had lower evidence quality ratings (taking an exam in a tidy versus untidy classroom). Additionally, although evidence quality was correlated with decisions for both the tidy classroom and exercise interventions, participants’ likelihood of implementing was significantly higher than their evidence quality ratings only for the tidy classroom intervention. Follow-up analyses revealed that participants were more likely to notice methodological flaws in the study about the tidy classroom intervention than the exercise intervention, and that the intervention effect on incorporate decisions was mediated by participants’ flaw detection rates. At the same time, we found that participants were more likely to reference their personal experiences and beliefs when explaining their decision to implement the tidy classroom. Consistent with these findings, in a separate sample of participants, we found that people had stronger prior beliefs about the tidy classroom intervention, which participants rated as both more plausible and more likely to reflect their own personal experience than the exercise intervention. Thus, despite acknowledging the flawed science behind the tidy classroom intervention, participants were generally more willing to implement the tidy classroom intervention in a hypothetical classroom setting, both because of its intuitive plausibility as an effective learning intervention and because it resonated more with participants’ personal experiences. This idea is summed up by the following participant explanation (a sentiment that was echoed by many other participants): “Though I find the study to be unconvincing, I think from personal experience that working in a clean environment is more productive than working in a messy one.” Thus, our findings from Experiment 1 suggest that people are capable of critically evaluating low quality evidence for belief-consistent ideas, but may underweigh low quality evidence when deciding whether to implement belief-consistent ideas.

Experiment 2 extended these findings to confirm that the plausibility of the intervention was the main driving factor in participants’ decisions and to test whether our results would generalize to contexts other than the tidy classroom and exercise interventions. Based on pretesting of prior beliefs about various learning interventions, we chose an additional *low plausible* intervention (napping to improve learning at school) and an additional *high plausible* intervention (using virtual reality to learn science). Consistent with our findings in Experiment 1, participants in Experiment 2 were more likely to implement the *high plausible* (tidy classroom and virtual reality) than *low plausible* interventions (exercise and napping). Additionally, decisions to implement the interventions were significantly mediated by prior beliefs about both the effectiveness and practicality of the interventions. Importantly, we again found that perceptions of evidence quality were dissociated from decisions, such that implementation likelihood ratings were greater than evidence strength ratings for the *high plausible* interventions but lower than evidence strength ratings for the *low plausible* interventions. We again confirmed that participants were more likely to mention prior beliefs and personal experience as the basis for their decision to implement *high plausible* interventions than *low plausible* interventions; additionally, participants were more likely to reference prior beliefs than the study itself as the basis for their decision, but only for the *high plausible* interventions.

One important difference between the two experiments was that while evidence strength ratings were lower for the more plausible intervention in Experiment 1, evidence strength ratings were higher for the more plausible interventions in Experiment 2. Additionally, whereas participants were more likely to identify specific methodological flaws in the more plausible study in Experiment 1, there were no differences in flaw detection rates for *high* versus *low plausible* interventions in Experiment 2. Thus, it is unclear why participants rated the *high plausible* interventions as having greater evidence quality in Experiment 2. One possibility is that they did not evaluate the evidence as critically because of their prior beliefs (e.g., due to confirmation bias, or because they evaluated the studies superficially rather than analytically). Additionally, the participant samples differed between the two experiments, with an undergraduate sample in Experiment 1 and Prolific participants in Experiment 2; thus, there may be baseline differences in propensities to critically evaluate scientific evidence between these two samples. However, the fact that we still observed a dissociation between perceived evidence quality and decisions to implement the learning interventions (for both *low* and *high* plausible interventions) in Experiment 2 suggests that evidence quality was again underweighed as a decision factor.

The choice of an educational context for the present studies was deliberate. There is oft- repeated despair that the field of education (not just teachers, but policy makers, administrators, and the general public) relies more on personal beliefs and anecdotes than science (Halpern, 2005; Robinson & Levin, 2019; Seidenberg, 2017). As Halpern argues, much of the fault lies in science communication. A better understanding of how best to communicate the science of education, such that stakeholders will consider and also critically evaluate science-based recommendations, is crucial. However, increasing critical evaluation of evidence alone may not be sufficient, as our studies suggest that making decisions about highly plausible learning interventions overrides low-quality evidence as a factor in hypothetical implementation decisions. Here, we presented participants with conclusions that were not supported by the evidence. Indeed, such examples are relevant in the real world—many educational products are sold as being “evidence-based” or supported by neuroscience or cognitive science, though the evidence base may not actually support the claims made by the marketers. For example, many educators continue to incorporate the learning styles theory into their pedagogy, despite the consistent lack of evidence that teaching students in their preferred learning style improves learning (e.g., Nancekivell et al., 2020; Pashler et al., 2008). However, further research is also necessary to address the issue highlighted by Halpern (2005) and Seidenberg (2017)—how to convince stakeholders to rely on high quality evidence in the face of personal beliefs supporting a view not consistent with the science.

The present studies also provide proof of concept that under some conditions, there can be a mismatch between people’s evaluations of the quality of evidence and their ultimate decisions. People might recognize flaws in a study and nonetheless choose to implement the recommendations based on the study—particularly if those recommendations are consistent with their prior beliefs. People may also struggle to critically evaluate education studies in particular because of strong prior beliefs about the effectiveness of certain learning interventions. In a study investigating learning styles beliefs and teaching practices among college instructors, Newton and Miah (2017) found that 58% of instructors initially reported believing the learning styles theory. Even more striking was their finding that, after being informed about the lack of evidence for the learning styles theory, 46% of participants agreed with the statement “Even though there is no ‘evidence base’ to support the use of Learning Styles, it is my experience that their use in my teaching benefits student learning,” and 30% of participants responded that they would continue to use the learning styles theory in their teaching. Consistent with our own findings, the findings of Newton and Miah (2017) imply that, for many educators, there is a disconnect between their belief about the scientific support for a learning theory and their practice. Educators may persist in using the learning styles theory *despite* their awareness of the strong body of evidence against the effectiveness of learning style interventions, possibly due to positive personal experiences with implementing the learning styles theory.

The present results are limited by use of only four exemplar scenarios in a single context—educational achievement. Future research should systematically consider the conditions under which flawed evidence is nonetheless considered to support implementation decisions in different domains. Our focus in the present studies was to examine how the plausibility of an intervention influenced evaluation of flawed evidence and, ultimately, implementation likelihood. However, we did not explicitly test how other baseline conditions affect implementation likelihood, such as high quality science evidence (in the context of low or high plausibility), or no evidence (i.e., an assertion). Including the full set of possible conditions under a variety of controlled contexts is necessary for a more complete understanding of how scientific evidence and prior beliefs influence decision-making.

Another limitation is that participants in Experiment 2 may have been biased by our initial questions asking about their beliefs about the plausibility and practicality of the learning interventions. Although it was necessary to gather this prior belief data to test whether prior beliefs mediated implementation decisions, it is possible that simply asking participants to reflect on their prior beliefs before reading the articles influenced their evidence quality judgments and/or implementation decisions. Further work is necessary to test the extent to which prior belief assessments affect later critical analysis of evidence as well as evidence-based decisions.

A final limitation is that the implementation judgments used in our studies were hypothetical and perhaps not relevant to the participants in our study. To what extent might implementation decisions be influenced by anecdotes and prior beliefs when making actual decisions or at least hypothetical decisions that might be more relevant to the participants? It is possible that individuals with more domain knowledge are generally more critical of evidence regardless of the presence of anecdotes; for example, teachers might be more likely to consider the possibility that coming from a math class to take a math test could present a confound, and they could weigh the flaws more heavily in their implementation judgments. On the other hand, given the findings of Newton and Miah (2017) and our own findings, teachers might persist in implementing an intervention even if they acknowledge that it is backed by flawed science, particularly if the intervention jibes with their own personal experience or the experiences of other instructors.

## Conclusion

In conclusion, our studies show that decisions to implement interventions backed by flawed scientific evidence are strongly influenced by prior beliefs about the intervention, particularly in regards to personal experience and plausibility. Moreover, identifying the flawed evidence behind the interventions was not enough to dissuade participants from implementing the interventions. This indicates a more general problem in people’s decision-making, namely that scientific evidence does not carry as much weight as it should in decisions that are supposedly ‘evidence-based.’ Many participants also mentioned that while the evidence was lacking for the learning interventions, there would be little cost to trying them out in a hypothetical classroom (e.g., “Even if the evidence supporting it is not convincing, there is no harm in having a tidy and organized classroom”). However, in many contexts, there is a lot to lose when people waste time and money on interventions that don’t work in lieu of ones that are actually effective, simply because of a failure to follow evidence-based recommendations appropriately.

## Data Availability

All data and materials are available upon request.

## Appendix 1

### Experiment 1 articles

#### Tidy classroom control (with descriptive text as a substitute for anecdote)

Some education specialists have argued that study environments can somehow influence a person's mood while studying. This could explain why a tidy room may have a positive effect on students’ academic performance. Based on this belief some teachers require students to keep their desks clean, hoping that having a tidy desk can help their students keep an organized mind. When children are at home, parents might help them clean the room, thus creating a cozy atmosphere. Tidiness at school has gradually become a requirement for most children, but whether or not tidiness can actually impact learning requires extensive research.

#### Exercise control

Language learning is interesting. As infants, almost all of us picked up our first language easily. We didn't have to be formally taught; we simply absorbed words and concepts. But as we enter the middle or high school, the brain generally begins to lose some of its innate language capability; it displays less growth in areas of the brain related to language. As a result, for most of us, it becomes harder to learn a second language.
